# Psychological crisis in patients with primary liver cancer after transarterial chemoembolization: epidemiological characteristics and associated factors

**DOI:** 10.1186/s12957-026-04217-6

**Published:** 2026-02-03

**Authors:** Shanshan Zhang, Ting Han

**Affiliations:** 1 Department of Interventional Radiology, The First Affiliated Hospital of Soochow University, Suzhou, Jiangsu province China; 2 Department of Stomatology, The First Affiliated Hospital of Soochow University, Suzhou, Jiangsu province China

**Keywords:** Transarterial chemoembolization, Psychological crisis, Cancer, Nursing, Care

## Abstract

**Background:**

Transarterial chemoembolization (TACE) remains a widely embraced therapeutic modality for primary liver cancer. The present study sought to explore the prevalence of psychological crisis in patients with primary liver cancer following TACE and pinpoint its associated factors, to provide actionable implications for enhancing clinical treatment and care protocols.

**Methods:**

A cross-sectional study design was utilized. Eligible participants were patients diagnosed with primary liver cancer who had undergone TACE and admitted to our hospital during the period from January 2023 to March 2025. The Triage Assessment Form (TAF) was adopted to assess the psychological crisis status of all enrolled patients.

**Results:**

A total of 276 primary liver cancer patients who had received TACE were included in the final analysis. The mean total score of psychological crisis among these patients was 18.25 ± 3.17. Spearman rank correlation analysis revealed significant associations between psychological crisis scores and age, gender, marital status, educational level, and average monthly household income (all *p* < 0.05). Multivariate linear regression analysis, adjusted for clinical covariates (Child-Pugh class, BCLC stage, TACE session frequency, post-procedural pain, and complications), confirmed the above five sociodemographic factors as independent correlates of psychological crisis scores (all *p* < 0.05). Collectively, these factors explained 56.2% of the variance in the outcome variable, with no statistically significant associations observed between psychological crisis scores and the included clinical variables (all *p* > 0.05).

**Conclusion:**

Patients with primary liver cancer following TACE exhibit moderate acute post-procedural psychological crisis-like states, which are primarily associated with sociodemographic vulnerability factors rather than objective clinical indicators. Targeted psychological screening and brief interventions for high-risk groups (younger patients, females, unmarried/divorced individuals, those with lower educational attainment, and lower household income) are critical to mitigate the adverse impacts of acute psychological distress on perioperative TACE care.

## Introduction

Primary liver cancer (PLC) is a highly prevalent malignant tumor with substantial global disease burden; in China, it ranks fourth among new cancer cases, posing significant challenges to public health [1–3]. Transarterial chemoembolization (TACE) is a first-line therapeutic modality for PLC, effectively controlling tumor progression and prolonging survival [4]. However, TACE patients face unique psychological stressors, including disease-related uncertainty, treatment side effects, repeated procedural trauma, and financial burden [5, 6]. These stressors often trigger acute psychological crisis-like states, which can impair treatment adherence and quality of life if unaddressed [7, 8].

Despite growing recognition of psychological distress in PLC patients post-TACE, existing research has focused on general prevalence rather than acute post-procedural psychological responses and their specific correlates. Critical gaps remain: sociodemographic factors’ influence on acute psychological states is understudied, and clinical variables (e.g., Child-Pugh class, BCLC stage, TACE session frequency) have not been integrated into analyses to clarify their confounding role. Additionally, interventions tailored to TACE-specific psychological stressors are lacking. To fill these gaps, this study aimed to evaluate the prevalence of acute post-procedural psychological crisis-like states in PLC patients post-TACE and identify associated factors (including sociodemographic and clinical variables), providing evidence for targeted perioperative care.

## Methods

### Study design

This study utilized a cross-sectional design to investigate the psychological distress in patients following TACE for primary liver cancer.

### Ethical consideration

The research protocol was reviewed and approved by the Institutional Ethics Committee of hospital (Approval No. 2025373) in compliance with the Declaration of Helsinki. Prior to participation, all eligible patients received detailed written and verbal explanations regarding the study objectives, procedures, potential risks, and benefits. Written informed consent was obtained from each participant, with documentation retained for verification. Clinical trial number: not applicable.

### Sample size calculation

In accordance with established guidelines from prior research [9], the recommended sample size for a questionnaire survey is typically 10 to 15 times the number of items included in the scale. In the present study, the scale comprises a total of 10 variables. To account for potential losses during scale distribution, as well as to ensure adequate effectiveness and minimize sampling error, a 10% loss rate was factored into the calculation. The sample size was thus computed as follows: (10* 15 * (1 + 10%)) = 165. Consequently, a minimum of 165 patients was required for this survey to achieve robust and reliable results.

### Study population

In this study, we employed a purposive sampling method to select patients with primary liver cancer who underwent TACE and were hospitalized in our hospital from January 2023 to March 2025. The choice of purposive sampling was guided by the need to focus on a specific patient population that met our inclusion criteria, ensuring that the sample was representative of individuals undergoing TACE for primary liver cancer within the defined time frame. The inclusion criteria were as follows: patients diagnosed with primary liver cancer through pathological or imaging examinations and who had undergone TACE treatment, aged ≥ 18 years, and had a clear understanding of their condition and voluntarily participated in this study. The exclusion criteria were as follows: patients with other malignant tumors or severe systemic diseases (such as heart failure, renal failure, etc.) in addition to primary liver cancer, patients who developed severe complications after TACE (such as hepatic encephalopathy, etc.) that prevented them from cooperating with the relevant assessments of the study, and patients who refused to participate in the study.

### Survey tool

We collected following information regarding the characteristics of patients with primary liver cancer: age, gender, body mass index (BMI), occupation, marital status, place of residence, educational level, average monthly household income, living arrangement, liver function classification (based on Child-Pugh score) and tumor progression stages (based on BCLC staging system), number of TACE sessions received, post-procedural complication status, and post-procedural pain scores measured via the Numeric Rating Scale (NRS).

In this study, the Triage Assessment Form (TAF) was employed to evaluate the psychological crisis status of patients. TAF, a tool designed by Myer et al. in 1992, is utilized to assess individuals’ responses to crisis events [10]. This tool was selected over alternative, widely used psycho-oncological scales (e.g., Self-Rating Anxiety Scale [SAS], Self-Rating Depression Scale [SDS], Distress Thermometer [DT]) for three key reasons. First, unlike SAS and SDS, which focus exclusively on single emotional states (anxiety or depression), the TAF comprehensively evaluates reactions across three core domains: affective, cognitive, and behavioral—aligning with the multifaceted psychological stress responses triggered by TACE. Second, compared with the DT, a single-item scale with limited dimensionality that only quantifies the severity of general distress, the TAF enables detailed assessment of crisis-related impairments, which is critical for identifying targeted intervention needs. Third, the TAF was originally developed for acute crisis scenarios, which matches the study’s focus on psychological responses within 24 h post-TACE—a period characterized by acute physical discomfort, treatment-related uncertainty, and situational stress.

This comprehensive instrument evaluates reactions across three dimensions: affective, cognitive, and behavioral. The affective domain assesses emotional responses, such as the congruence of emotions with the situation and emotional stability. The behavioral domain observes behavioral reactions, including the effectiveness of coping behaviors and the presence of any abnormal behaviors. The cognitive domain analyzes cognitive responses, such as perception and interpretation of the crisis event, as well as the clarity of thought processes.

The assessment tool utilized in this study comprises six dimensions, each consisting of six items. Scoring for each item is determined based on the degree of impairment, ranging from no impairment (1 point) to severe impairment (10 points), with intermediate levels categorized as mild (2–3 points), low (4–5 points), moderate (6–7 points), and high (8–9 points) impairment [11]. A total score of 12 or higher indicates the presence of a psychological crisis. Notably, while the TAF has demonstrated robust psychometric properties and extensive utility in general clinical psychological assessments and crisis interventions [12, 13], it had not been validated in the primary liver cancer post-TACE population prior to this study.

The Chinese patient version of the TAF utilized in this study was adapted from the original English scale in strict accordance with internationally recognized cross-cultural validation guidelines, with detailed protocols referenced in the revised manuscript. The cross-cultural adaptation process comprised five rigorous steps: (1) Two independent bilingual researchers, with professional backgrounds in clinical psychology and hepatology, performed forward translation of the original English version into Chinese; (2) A third researcher, who was blinded to the original scale, synthesized the two forward translations to generate a preliminary Chinese version; (3) Two additional bilingual experts conducted back-translation of the preliminary Chinese version into English; (4) A multidisciplinary research team, consisting of clinical psychologists, hepatologists, and research methodologists, compared the back-translated version with the original English scale, resolved semantic inconsistencies, and refined the scale to form the final Chinese patient version; (5) A pilot pre-survey was conducted among 20 primary liver cancer patients who had undergone TACE to evaluate the item clarity and content acceptability of the adapted scale, with no ambiguous or inappropriate items identified. Written copyright permission for the use of this scale was obtained from the copyright holder prior to the initiation of formal data collection.

To verify the psychometric properties of the adapted TAF in our target population, we further conducted reliability and validity analyses on the formal survey data. Reliability testing revealed that the overall Cronbach’s α coefficient of the scale was 0.82, with Cronbach’s α coefficients for the affective, cognitive, and behavioral domains ranging from 0.78 to 0.81, indicating good internal consistency reliability. Construct validity was assessed via confirmatory factor analysis (CFA), which yielded acceptable model fit indices (χ²/df = 2.13, RMSEA = 0.06, CFI = 0.91, TLI = 0.90), supporting the three-factor structure of the adapted TAF in primary liver cancer patients post-TACE. These findings confirm that the Chinese version of the TAF is a reliable and valid tool for evaluating psychological crisis responses in this specific patient cohort.

### Survey process

In our study, the administration of the questionnaires was meticulously planned to ensure data accuracy and reliability. Trained nurses, who had undergone a standardized training session prior to data collection, were responsible for administering the questionnaires. This training was specifically designed to ensure consistency in the administration process and to familiarize the nurses with the study’s specific requirements, thereby minimizing potential biases and procedural errors. To capture the patients’ psychological and physiological responses immediately following surgery, the survey was conducted within 24 h postoperatively. This timing was chosen to provide a timely and accurate assessment of the patients’ psychological state, enabling a more precise evaluation of their level of psychological crisis. Before the survey began, the investigator engaged in thorough communication with each patient. The study’s objectives and significance were explained in detail to ensure that the patient fully understood the research content. The investigator then assured the patient that all data would be strictly confidential, used solely for scientific research purposes, and that no personal information would be disclosed. Following this, the patient was required to sign an informed consent form, indicating their voluntary participation in the study. After obtaining consent, the investigator provided a detailed explanation of the questionnaire completion methods. Patients were required to complete the questionnaire independently to maintain the authenticity and objectivity of the data. The completion time was set at 30 min, and upon completion, the investigator collected the questionnaire immediately to ensure data integrity and timeliness.

### Data analysis

Data analysis was conducted using SPSS software (version 25) to ensure rigorous and standardized statistical processing. Prior to analysis, all data underwent thorough distribution testing to verify their characteristics and suitability for subsequent analyses. For categorical variables, descriptive statistics were generated and presented as percentages to reflect the proportions of different categories within the sample. Continuous variables, which were confirmed to follow a normal distribution, were summarized using the mean ± standard deviation notation. To evaluate the association between psychological crisis scores and patient characteristics in individuals with primary liver cancer following TACE, Pearson correlation analysis was employed. The associated factors of patient fatigue were identified through multivariate linear stepwise regression analysis. Statistical significance was set at a p-value of less than 0.05.

## Results

A total of 276 patients with primary liver cancer after TACE were included. As indicated in Table 1, the mean age of the patients was 58.46 ± 8.40 years, with the majority being male (73.19%). The average BMI was 21.06 ± 1.85, and the majority of patients resided in urban areas (65.94%). The majority of patients were classified as Child-Pugh Class A (54.3%), indicating relatively well-preserved liver function. In terms of tumor progression, the predominant stage was BCLC Stage A (29.0%), which is characterized by early-stage disease with favorable prognostic features.

**Table 1:** The characteristics of patients with primary liver cancer (*n* = 276)

Characteristic	Cases	Percentage
Age (years)		
< 60	149	54.0%
≥ 60	127	46.0%
Gender		
Male	202	73.2%
Female	74	26.8%
BMI (kg/m²)		
< 24	215	77.9%
≥ 24	61	22.1%
Occupation		
Industrial Worker	65	23.6%
Farmer	50	18.1%
Self-employed	69	25.0%
Civil Servant	29	10.5%
Retired	38	13.8%
Other	25	9.1%
Marital status		
Married	231	83.7%
Unmarried	16	5.8%
Divorced	20	7.2%
Widowed	9	3.3%
Place of residence		
Rural area	94	34.1%
Urban area	182	65.9%
Educational level		
Elementary school	62	22.4%
Junior High school	89	32.2%
Senior High school	65	23.6%
Vocational school	35	12.7%
College or University	25	9.1%
Average monthly household income		
< 5000	184	66.7%
≥ 5000	92	33.3%
Living arrangement		
Living alone	36	13.1%
Living with spouse	198	71.7%
Living with children	28	10.1%
Other	14	5.1%
Liver function classification (Child-Pugh score)		
Class A	193	70.0%
Class B	64	23.2%
Class C	19	6.9%
Tumor progression stages (BCLC staging system)		
BCLC Stage 0	75	27.2%
BCLC Stage A	102	37.0%
BCLC Stage B	54	19.6%
BCLC Stage C	32	11.6%
BCLC Stage D	13	4.7%
Number of TACE sessions		
1	156	56.5%
2	82	29.7%
≥ 3	38	13.8%
Post-procedural complication status		
No complications	219	79.3%
With complications	57	20.7%
Post-procedural pain score (NRS)		
0–3 (mild pain)	124	44.9%
4–6 (moderate pain)	98	35.5%
7–10 (severe pain)	54	19.6%

*BMI* body mass index, *BCLC* Barcelona Clinic Liver Cancer, *NRS* Numeric Rating Scale

The total score for acute post-procedural psychological crisis-like states in patients following liver cancer TACE was 18.25 ± 3.17 points (Table 2). Among the three core domains of the Triage Assessment Form (TAF), the affective domain had the highest score (6.80 ± 1.29), followed by the cognitive domain (6.62 ± 1.20), with the behavioral domain having the lowest score (6.49 ± 1.24). Based on these domain scores, patients with liver cancer following TACE experienced moderate impairment in acute post-procedural psychological crisis responses.

**Table 2:** Psychological crisis score in patients with primary liver cancer following TACE (*n* = 276)

Item	Average score (standard deviation)
Affective domain	6.80 ± 1.29
Cognitive domain	6.62 ± 1.20
Behavioral domain	6.49 ± 1.24
Total score	18.25 ± 3.17

As revealed in Table 3, Spearman rank correlation analysis (instead of Pearson correlation analysis, to account for the categorical nature of key variables) indicated that age (*r* = 0.590, *p* = 0.012), gender (*r* = 0.623, *p* = 0.016), marital status (*r* = 0.571, *p* = 0.040), educational level (*r* = 0.604, *p* = 0.022), and average monthly household income (*r* = 0.616, *p* = 0.009) were significantly associated with psychological crisis scores in patients with primary liver cancer following TACE. No significant correlations were observed between psychological crisis scores and BMI, occupation, place of residence, living arrangement, Child-Pugh class, or BCLC stage (all *p* > 0.05).

**Table 3:** Spearman rank correlation analysis of the association between psychological crisis scores and patient characteristics in patients with primary liver cancer following TACE (*n* = 276)

Variable	ρ	*p*-value
Age	0.590	0.012
Gender	0.623	0.016
BMI	0.114	0.168
Occupation	0.107	0.093
Marital status	0.571	0.040
Place of residence	0.185	0.102
Educational level	0.604	0.022
Average monthly household income	0.616	0.009
Living arrangement	0.087	0.106
Liver function classification	0.250	0.058
Tumor progression stages	0.189	0.070
Number of TACE sessions	0.121	0.154
Post-procedural complication status	0.135	0.127
Post-procedural pain score (NRS)	0.142	0.113

*BMI* body mass index, *NRS* Numeric Rating Scale

Table 4 presents the results of the multivariate linear regression analysis, which incorporated Child-Pugh class, BCLC stage, number of TACE sessions, post-procedural complication status, and NRS pain scores as covariates to control for potential confounding effects. The analysis identified five independent correlates of psychological crisis scores in this patient cohort: age (β = -0.101, *p* = 0.016), gender (β = 0.115, *p* = 0.008), marital status (β = 0.203, *p* = 0.013), educational level (β = 0.091, *p* = 0.020), and average monthly household income (β = -0.264, *p* = 0.005). Notably, none of the included clinical variables showed statistically significant associations with psychological crisis scores (all *p* > 0.05). After adjusting for clinical covariates, the five sociodemographic factors collectively explained 56.2% of the variance in psychological crisis scores (adjusted R²=0.562, F = 21.842, *p* < 0.001). The β coefficients have clear clinical interpretations: for example, a 1-year increase in age was associated with a 0.756-point decrease in TAF total score, while female gender was associated with a 1.239-point higher TAF score compared with male gender; a 1–2 point change in TAF score is considered clinically meaningful in acute crisis assessment.

**Table 4:** Multivariate linear regression analysis of factors associated with psychological crisis in patients with primary liver cancer following TACE (*n* = 276)

Variables	B	β	t-value	*p*-value	VIF
Constant	8.605	-	2.40	0.017	-
Age	-0.756	-0.101	-1.84	0.016	1.24
Gender	1.239	0.115	2.19	0.008	1.18
Marital status	1.977	0.203	2.80	0.013	1.31
Educational level	4.384	0.091	3.24	0.020	1.27
Average monthly household income	-1.945	-0.264	-2.96	0.005	1.19
Child-Pugh class	0.321	0.045	0.89	0.375	1.42
BCLC stage	0.412	0.058	1.03	0.304	1.38
Number of TACE sessions	0.287	0.039	0.76	0.448	1.22
Post-procedural complication status	0.354	0.049	0.92	0.359	1.16
Post-procedural pain score (NRS)	0.295	0.042	0.81	0.419	1.25

Adjusted R²=0.562, F = 21.842, *p* < 0.001

*VIF* Variance Inflation Factor, *BCLC* Barcelona Clinic Liver Cancer, *NRS* Numeric Rating Scale, *VIF* < 3 indicates no significant multicollinearity

## Discussion

### Prevalence of acute post-procedural psychological crisis-like states

This study found that PLC patients post-TACE had a mean TAF score of 18.25 ± 3.17, indicating moderate acute post-procedural psychological impairment. Among the three domains (affective, cognitive, behavioral), the affective domain had the highest score, consistent with previous observations that emotional distress is the most prominent psychological response to acute medical stress [14–16]. Notably, the study cohort was predominantly composed of patients with well-preserved liver function (Child-Pugh Class A, 70.0%) and early-stage tumors (BCLC Stage 0/A, 64.2%), yet still exhibited significant acute psychological distress. This highlights that even patients with favorable clinical profiles are vulnerable to post-TACE psychological stress, emphasizing the need for routine psychological screening regardless of disease severity.

### Associated factors and TACE-specific mechanisms

Multivariate linear regression analysis, adjusted for clinical covariates (Child-Pugh class, BCLC stage, TACE session frequency, post-procedural pain, and complications), confirmed that age, gender, marital status, educational level, and average monthly household income are independent correlates of acute psychological crisis-like states (all *p* < 0.05). These sociodemographic factors collectively explained 56.2% of the variance in TAF scores, and their associations remained stable after controlling for clinical variables—indicating their robust associations on acute post-procedural psychological responses.

The β coefficients have clear clinical significance: a 1-year increase in age was associated with a 0.756-point decrease in TAF score (β = -0.101), and female gender was associated with a 1.239-point higher TAF score than males (β = 0.115). Since a 1–2 point change in TAF score is clinically meaningful in acute crisis assessment [10, 17], these associations are not only statistically significant but also clinically relevant. The observed vulnerability patterns (younger age, female gender, unmarried/divorced status, lower education, lower income) align with general psycho-oncology research, but their mechanisms are amplified in the TACE context: younger patients may face greater anxiety about long-term survival, career disruption, and family responsibilities; females’ heightened emotional sensitivity may be exacerbated by procedural pain and treatment uncertainty; unmarried/divorced patients lack stable spousal support, a key buffer against acute medical stress; and lower education/income limits access to treatment information and financial resources, intensifying helplessness regarding TACE’s repeated cycles and associated costs [18–20].

Notably, no significant associations were found between psychological crisis scores and clinical variables (Child-Pugh class, BCLC stage, TACE sessions, post-procedural pain/complications; all *p* > 0.05). This unexpected finding suggests that acute psychological responses within 24 h post-TACE are more strongly driven by sociodemographic vulnerabilities than objective disease severity or immediate treatment-related factors. This may be because acute post-procedural distress is primarily triggered by the invasive procedure itself and associated uncertainty, rather than pre-existing clinical status. Future studies should explore whether clinical variables exert greater influence on long-term psychological outcomes.

### Targeted intervention strategies for TACE patients

Based on the findings, we propose TACE-specific, multi-level interventions to mitigate acute psychological distress, eschewing generic policy recommendations: at the individual level, targeted psychological screening using the TAF should be implemented within 24 h post-TACE for high-risk groups (younger patients, females, unmarried/divorced individuals, and those with lower educational attainment or household income), with immediate brief interventions (e.g., emotional validation, cognitive restructuring to address treatment uncertainty) provided to patients with high TAF scores; at the clinical level, interventional radiologists and nurses should receive training to recognize acute post-procedural psychological responses, and pain management (to alleviate procedural discomfort) should be integrated with psychological support into perioperative care, including pre-procedural counseling on anticipated stressors and coping strategies; and at the institutional level, multidisciplinary teams comprising hepatologists, interventional radiologists, clinical psychologists, and social workers should be established to deliver comprehensive care, TACE-specific patient support groups set up to facilitate peer support, and financial counseling services offered to reduce the economic burden associated with repeated procedures [21–24].

### Limitations

This study acknowledges several limitations that warrant careful consideration when interpreting the findings: it adopted a single-center, purposive sampling strategy and cross-sectional design, where the use of purposive sampling of inpatients may have introduced selection bias by excluding patients with severe complications, sedation, intense pain, or other conditions precluding cooperation—potentially underrepresenting individuals with the highest psychological burden and underestimating the prevalence of acute post-procedural psychological crisis-like states—while the single-center nature limits geographical and population representativeness, restricting the generalizability of findings to the broader population of primary liver cancer patients undergoing TACE; the absence of a control group hinders definitive attribution of observed psychological responses to TACE itself or confounding factors (e.g., hospital environment, acute post-procedural discomfort); although we validated the Chinese version of the Triage Assessment Form (TAF) in our target population, the original TAF lacked prior validation in liver cancer patients undergoing TACE, and while internal psychometric analyses confirmed acceptable reliability and construct validity, this lack of specific clinical validation may impact score interpretation, with the TAF also potentially failing to capture all dimensions of relevant psychological distress; psychological status was only assessed once within 24 h post-TACE, providing merely a “snapshot” of acute post-procedural psychological responses (rather than general or long-term psychological crisis status) and confounding these responses with factors such as immediate post-interventional pain, anxiety about complications, and the hospital environment, without longitudinal follow-up (e.g., 1 week, 1 month post-TACE) to explore the trajectory or persistence of distress; and finally, the study focused solely on sociodemographic and selected clinical characteristics, neglecting other potentially influential factors (e.g., social support, concurrent medical conditions, pre-intervention quality of life, prior psychological history) that may confound the association between TACE and acute psychological responses. Future research should address these limitations through multicenter designs with random sampling, inclusion of control groups, further validation or development of tailored assessment tools, longitudinal follow-up measurements, and incorporation of additional confounding variables to enhance the external validity, analytical power, and comprehensiveness of findings.

## Conclusion

This single-center cross-sectional study provides preliminary evidence that PLC patients exhibit moderate acute post-procedural psychological crisis-like states within 24 h of TACE. These states are independently associated with sociodemographic factors (age, gender, marital status, educational level, household income) rather than clinical variables (e.g., disease stage, liver function). Targeted psychological screening and TACE-specific interventions for high-risk groups may improve perioperative care. Due to study limitations, causal inferences cannot be drawn; future multicenter longitudinal studies with validated tools are needed to confirm the findings and explore the long-term trajectory of psychological distress in this population.

## Data Availability

The data associated with the paper are not publicly available but are available from the corresponding author on reasonable request.

## Abbreviations

TACE: Transarterial chemoembolization
IARC: International Agency for Research on Cancer
WHO: World Health Organization
BMI: body mass index
